# Effects of a Need-Supportive Motor Skill Intervention on Children’s Motor Skill Competence and Physical Activity

**DOI:** 10.3390/children7030021

**Published:** 2020-03-17

**Authors:** Joonyoung Lee, Tao Zhang, Tsz Lun (Alan) Chu, Xiangli Gu

**Affiliations:** 1 Department of Kinesiology, Health Promotion and Recreation, University of North Texas, Denton, TX 76203, USA; joonyounglee@my.unt.edu; 2 Department of Psychology, University of Wisconsin-Green Bay, Green Bay, WI 54311, USA; chua@uwgb.edu; 3 Department of Kinesiology, The University of Texas at Arlington, Arlington, TX 76019, USA; Xiangli.Gu@uta.edu

**Keywords:** self-determination theory, need-supportive teaching, fundamental motor skills, motor skill intervention, physical activity, children

## Abstract

A need-supportive environment can provide various motivational benefits to impact children’s psychomotor developmental levels. However, very little is known about the effects of need-supportive motor skill intervention on children’s motor skill competence and physical activity by gender. Guided by self-determination theory (SDT), this study aimed to (a) investigate the effect of a need-supportive fundamental movement skill (FMS) program on children’s FMS competence and moderate-to-vigorous physical activity (MVPA), and (b) explore potential gender differences in these effects. Thirty-six children (63.8% girls; *M*_age_ = 6.52 ± 0.97) participated and were divided into two groups: an intervention group (24 need-supportive FMS sessions over eight weeks) and a control group. A repeated-measures multivariate analysis of variance (MANOVA) was used to examine the influence of the motor skill intervention on FMS competence and MVPA over time by group (intervention, control) and gender (boys, girls). The results showed (a) significant group differences between the intervention and control group in FMS competence and MVPA (*p* < 0.001), (b) non-significant gender differences between boys and girls in FMS competence and MVPA (*p* = 0.85), and (c) non-significant interaction effects over time (*p* = 0.52). The findings highlight that a need-supportive FMS program may enhance FMS development and daily physical activity for both genders during the early school years.

## 1. Introduction

National standards and grade-level outcomes emphasize fostering the maturation of fundamental motor skills (FMS) and developing the understanding of movement concepts in early grades [1]. FMS consist of two main categories: locomotor skills involving the movement of the body via space (e.g., running and galloping) and object control skills requiring hands and feet to manipulate an object (e.g., throwing and catching) [2]. FMS underscore specific and complex motor skills in varied activities across the lifespan [3]. Previous studies have demonstrated that sufficient FMS competence is associated with increased physical activity and improved health status over one’s lifetime [4,5,6]. These findings provide us with strong rationales to develop FMS competence in young children.

The importance of developing FMS competence among children has been emphasized by policy makers [7]; however, more than half of children lack adequate FMS competence when they exit elementary school in the United States [8,9], the United Kingdom [10], and Australia [11]. Children’s physical activity during school hours have consistently decreased due to reduced allotted time and low-quality instruction in physical education (PE) [9,12]. This trend results in less than half of students achieving the recommendation of 60 min of moderate-to-vigorous physical activity (MVPA) daily [7]. Recent literature reviews also emphasized that the importance of FMS competence served as a prerequisite for physical activity engagement throughout childhood, which will impact on physical activity later in adolescence [13,14,15]. Thus, it is essential to intervene in the development of children’s FMS competence, which is interconnected with children’s physical activity [4,16].

Previous studies have identified gender differences in FMS competence throughout childhood [17,18,19], although these studies have reported contradictory findings. Cliff et al. [20] demonstrated that girls showed higher levels of locomotor skills, such as running, galloping, and jumping, than boys (aged 3–5 years). However, other studies revealed that boys outperformed girls in locomotor skills in 3- to 5-year-old children [21] or in both locomotor and object control skills among 4- to 11-year-olds [22], while some studies found no gender differences in locomotor skills in children from 3 to 5 years old [23]. Identifying gender differences in FMS competence is needed to establish practical settings and equal opportunities for boys and girls to optimally develop FMS competence [19,20].

Gender differences in FMS competence can be elucidated by the interaction between environmental and biological factors [2]. It is imperative to consider proper instructional approaches to offer equal opportunities to enhance FMS competence in both genders of children [24]. A longitudinal study conducted by Barnett et al. [25] indicated that acquiring object control skills before 10 years of age for girls would be significant to enhance FMS competence across the lifespan. The findings also reported that girls might not receive enough instruction and sufficient opportunity to practice motor skills; thus, it could be important to provide particular motor skill interventions with instructional strategy to engage girls in learning FMS during early childhood [25].

Some studies have examined the effects of instructional climates to promote motivational learning in FMS intervention settings [26]. The children in the motivational climate group showed positive improvements in FMS competence compared to their counterparts in a less supportive instructional group [27,28]. Although previous studies have shown promising improvements in FMS competence among children 4 to 10 years old by changing the instructional climates as motivational environments, the lack of description about “when” and “where” was indicated [27]. The self-determination theory (SDT; [29]) explains people’s motivational and health choices, and supports the three basic psychological needs—autonomy, competence, and relatedness—as pivotal elements in one’s behavioral and psychological adjustment. *Autonomy* refers to the experience of free will in one’s own actions when engaging in an activity; *competence* is the feeling of confidence and effectiveness about an activity; and *relatedness* is a sense of belonging and closeness to others in an activity [29]. Given that students’ learning can be magnified in an efficient learning environment [30,31], it would be imperative to create a need-supportive environment (i.e., autonomy support, competence support, and relatedness support) in the development of FMS competence with efficient instructional strategies.

Guided by SDT, in this study we implemented the FMS intervention with need-supportive instruction in an afterschool program. Recently, Johnson et al. [32] implemented a motor skill program integrated with mastery motivational climates (stemming from SDT) during preschool playground time and demonstrated significant improvements in FMS competence. Nevertheless, these previous studies focused only on autonomy support (one of the three supports for basic psychological needs) to create a motivational climate. To date, very little is known about the effects of need-supportive motor skill interventions (supporting all three basic psychological needs for autonomy, competence, and relatedness) on children’s FMS competence and physical activity behavior by gender in elementary school settings. The purpose of this study, therefore, was twofold: (1) to investigate the effect of an 8-week, need-supportive, FMS-based afterschool program on children’s FMS competence and physical activity behavior; and (2) to explore potential gender differences (boys vs. girls) regarding the intervention effects.

## 2. Materials and Methods

### 2.1. Participants

The participants in the study were 38 K–2nd grade children from three public elementary schools in southwestern United States. However, two children’s data in the control group were excluded because they had transferred to other schools; hence, the final participants included 36 children (63.8% girls; M_age_ = 6.52 ± 0.97; age range 5–8 years). Specifically, based on the purpose of the intervention program, the schools provided lists of children who participated in the regular afterschool programs and recruitment flyers and consent forms were sent out to their parents to recruit participants randomly. Power analysis was performed using G*Power 3.1 (Faul et al., 2007 [33]), suggesting that 32 participants would be sufficient for 80% power (α = 0.05, effect size 0.3) to test the main outcomes (i.e., FMS competence and physical activity behavior). Approval to conduct the study was obtained from the Institutional Review Board, and parental informed consent forms and child assent forms were received in accordance with the participating school district and the Declaration of Helsinki before starting the study. (Project identification code: 16-357).

### 2.2. Intervention Procedure

**Figure 1:**
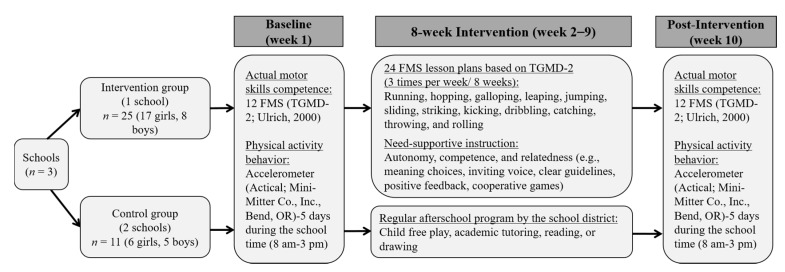
Overview of process and timeline of the 8-week, need-supportive fundamental motor skills (FMS) intervention. Note: TGMD-2 = Test of Gross Motor Development, 2nd edition.

Figure 1 presents an overview of the process and timeline of the intervention. Based on the school district’s suggestion and accessibility, researchers assigned participants at the school level to one of two conditions: the intervention (one school, *n* = 25) or control group (two schools, *n* = 11). Children in the intervention group (17 girls, eight boys) were assigned to the 8-week, FMS-based afterschool program (3:30 p.m. to 4:30 p.m.; 60 min) for three times per week, resulting in 24 sessions with need-supportive instruction (see Table 1). The control group (six girls, five boys) followed a regular afterschool program (e.g., unsupervised child free-play, academic tutoring). The typical afterschool programs (3:00 p.m. to 6:00 p.m.) in the schools did not provide any motor skill-related instructions. All three participating schools provided 10 min daily brain activity before class, 50 min physical education (3 days/week), 30 min daily recess, and 20 min outdoor activity during lunchtime daily (8:00 a.m. to 3:00 p.m.).

The 24-session intervention program was designed to teach the 12 basic motor skills (i.e., running, hopping, galloping, leaping, jumping, sliding, striking, kicking, dribbling, catching, overhand throwing, and underhand rolling) from the Test of Gross Motor Development-2 (TGMD-2; Ulrich, 2000 [34]). Each session included one introductory activity and two developmental activities based on developmentally age-appropriate, fun PE games (Please see Lee et al., 2020 [35]). In order to promote intrinsic motivation for the children to engage in the activities, the program was delivered using need-supportive instruction (e.g., autonomy, competence, and relatedness support) based on SDT [29]. To systematically develop the need-supportive instruction on the structured FMS-based afterschool program, the principal investigator and researchers (physical activity specialists and PE instructors for university-level students) held multiple training sessions to identify and categorize need-supportive instruction (see Table 1) delivered by other graduate research assistants. Additionally, we used the field observations and had weekly meetings to discuss and improve the quality control of need-supportive instruction once a week during the intervention period.

Each intervention session consisted of 60 min divided into three parts: (a) 10 min of warming-up preparation for FMS lessons and activities, (b) 45 min of focused FMS practices, and (c) 5 min of a closing activity. Two well-trained graduate research assistants, with more than three years of physical activity teaching experience, led the intervention sessions.

During teaching and preparation, we provided the children with specific instruction and demonstration concerning essential elements of performing each specific FMS skill (e.g., running: “Do you know how we can run faster? We use arm swinging. First, bend your elbows at 90 degrees and imagine you are lightly gripping small balls in each hand, then pump your arms fast”).

During the 45-min FMS practices, children were assigned into two groups based on low and high motor skill competence evaluated from their FMS performance in the first 10 min. Each graduate research assistant supervised each group (low and high motor skill competence groups) and provided the children with instructional feedback/reinforcement about their effort (e.g., “you practiced your kicking which is what you learned from today’s lesson; you did a great job of placing your non-kicking foot close to the ball when you kick the ball”). We also provided learning cues during each session, such as “arm swing”, “arm–leg opposition”, “knee-high”, and “bend leg” in the running session to keep reminding the children of the purpose of the activities [36].

During the 10-min closing activity, we asked the children what and how they learned FMS for the review of the skill (e.g., jumping: “What was our new movement skill today?” “How can we jump higher and further?” “What should you remember about your arms and knees in preparation for jumping?”), and performed a series of stretches to cool down.

### 2.3. Instrumentation

#### 2.3.1. Height and Weight

Participants’ height and weight (without shoes) were recorded by trained research assistants using a Health-o-Meter 500 KL digital physician height/weight scale (Pelstar, LLC, Countryside, IL, USA). Body mass index (BMI) was calculated using the following formula: (weight [kg]/height^2^ [m^2^]).

#### 2.3.2. Actual Fundamental Motor Skills (FMS) Competence

The TGMD-2 (Ulrich, 2000 [34]) was used to measure children’s actual FMS competence in two sections: six locomotor skills (i.e., galloping, hopping, leaping, running, horizontal jumping, sliding) and six object control skills (i.e., dribbling, catching, kicking, underhand rolling, striking, overhand throwing). Two well-trained graduate research assistants, who had completed three training sessions using TGMD-2, rated all FMS assessments. Before the measurement, both examiners established 90% interrater reliability through YouTube videos of children undertaking the TGMD-2 protocol and achieved 91% interrater reliability (0.89 locomotor skills and 0.92 object control skills) in the actual measurement. The FMS test was administered in each school’s indoor gymnasium with the school administrator’s permission. Individual FMS was measured twice for each skill using 3–5 criteria for a total duration of about 20–25 min. For each child, each motor skill was evaluated and marked as either *present* (1) or *absent* (0) based on performance criteria. Two subscales (locomotor and object control skills) were calculated from the sum of raw scores in each subset. Previous research using TGMD-2 showed high test–retest reliability (*r*s > 0.85) and good internal consistency (locomotor α = 0.85 and object control α = 0.88; [34]) for children’s FMS (3–11 years old). This study indicated high internal consistency for locomotor (α = 0.92) and object control skills (α = 0.97).

#### 2.3.3. Physical Activity Behavior

Children’s MVPA was analyzed to measure children’s physical activity behavior during school hours [49]. With the help of the trained graduate research assistants, the participants wore water-resistant accelerometers (Actical; Mini-Mitter Co., Inc., Bend, OR, USA) on an elastic band on their non-dominant hand for five consecutive days during the elementary school hours (8:00 a.m. to 3:00 p.m.). Data were selected in 60-s epoch lengths to detect spontaneous MVPA in the participants’ schools. The cutoff points of Activity Energy Expenditure (AEE) were sedentary behavior (AEE < 0.01 kcal·kg^−1^·min^−1^), light physical activity (0.01 ≤ AEE < 0.04 kcal kg^−1^·min^−1^), and MVPA (AEE ≥ 0.04 kcal kg^−1^·min^−1^) [50]. Accelerometer data were downloaded into Excel files using ActicalReader and Actcal Software 3.12 (Koninklijke Philips Electronics N.V., Amsterdam, The Netherlands). Due to the practical limitations, such as missing devices and participant compliance, the children wore the accelerometers only at school and had the devices removed at 3:00 p.m. with the research assistants’ guidance. Accelerometers provided objective information about the frequency, intensity, and duration of movement [51]. In addition, the device has demonstrated high reliability and validity when measuring physical activity in children [52].

### 2.4. Data Analysis

This study used Windows SPSS 25.0 (IBM Corp., Armonk, NY, USA) for data analyses, with *p* < 0.05 for statistical significance. Before conducting data analysis, we checked missing data, normality, and outliers. Independent-sample *t*-tests were used to examine any group and gender differences prior to the intervention. A 2 × 2 multivariate analysis of variance (MANOVA) with repeated measures over time (pre-and posttests) was used to examine the effects of the FMS intervention on FMS competence and MVPA during school by group (intervention, control) and gender (girls, boys). Follow-up univariate analyses were conducted to examine any significant group and gender differences. A gain score, indicating an improvement, was calculated for each study variable in each group (post-test scores minus pre-test score). Partial η^2^ (eta squared) was used as an index of effect size (i.e., small = 0.01, medium = 0.09, and large = 0.25) of variance explained. We used Hedge’s *g* to include a correction for the small sample size (n = 36) and to investigate the effect size [53]. The criteria for Hedge’s *g* are ≥0.20 (small), ≥0.50 (medium) and ≥0.80 (large), indicating effect sizes for group differences [54].

## 3. Results

### 3.1. Baseline Descriptive Statistics between Groups by Gender

The descriptive statistics are presented in Table 2. The independent-sample *t*-tests demonstrated no statistically significant differences between the groups and the genders from the baseline, but a statistically significant difference between genders emerged in object control skills [*t*(34) = 2.65, *p* = 0.01].

### 3.2. Multivariate Analysis of Variance

**Figure 2:**
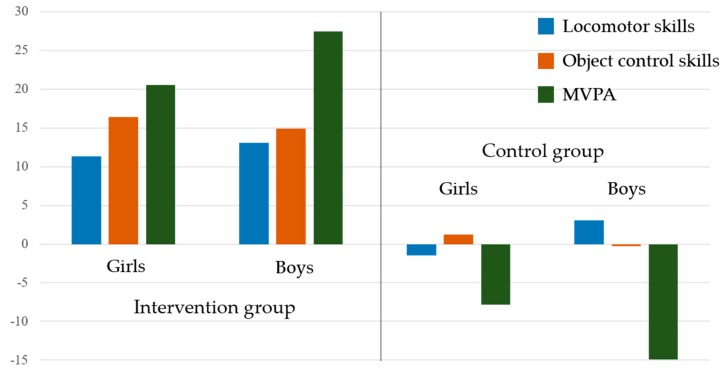
Gain scores in locomotor skills, object control skills, and moderate-to-vigorous physical activity (MVPA) by group and gender.

Table 3 indicates the means, standard deviations, and effect sizes (*g*) for the pre- and post-test scores on the FMS competence, MVPA, and gain scores. The repeated-measures MANOVA showed no significant interaction effects between group (intervention, control) and gender (girls, boys) on FMS competence and MVPA over time [*F*(2, 31) = 0.66, *p* = 0.52, Wilk’s Λ = 0.95, partial η^2^ = 0.04]. The follow-up univariate ANOVA showed significant group differences in locomotor skills [*F*(1, 34) = 4.13, *p* < 0.05, partial η^2^ = 0.11], object control skills [*F*(1, 34) = 10.81, *p* < 0.01, partial η^2^ = 0.24], and MVPA [*F*(1, 34) = 8.03, *p* < 0.01, partial η^2^ = 0.19] (see Table 3 and Figure 2). No statistically significant gender differences were found in locomotor skills [*F*(1, 34) = 0.14, *p* = 0.70, partial η^2^ = 0.01], object control skills [*F*(1, 34) = 2.56, *p* = 0.12, partial η^2^ = 0.07], and MVPA [*F*(1, 34) = 1.17, *p* = 0.28, partial η^2^ = 0.03]. The effect size was produced using Hedge’s *g* by comparing mean and standard deviation scores between the pre- and post-test and showed significant improvements on the children’s FMS competence and MVPA in the intervention group, with a medium to large effect size (range *g*s 0.49–1.92). Only boys in the control group demonstrated a small improvement in locomotor skills (*g* = 0.25), whereas their MVPA during school decreased significantly with a small effect size (*g* = 0.26). Boys and girls in the control group did not show any significant improvements in either FMS competence or MVPA compared to the intervention group (see Table 3 and Figure 2).

## 4. Discussion

### 4.1. Influences of a Need-Supportive Intervention on FMS Competence and Physical Activity Behavior during School

After the 8-week, need-supportive, FMS-based afterschool program, both boys and girls in the intervention group demonstrated greater improvements in FMS competence and MVPA during school time than those in the control group. The findings of significantly increased FMS competence through instructional climate change in the FMS intervention are in accordance with the findings of previous studies [27,32,55], which reflect the benefits of promoting a motivational climate in FMS intervention on both locomotor and object control skills among children compared to the control group. Boys in the unsupervised free play without any motor skill instruction showed minimum improvements in locomotor skills, but did not show any change in object control skills. Similar to the results of this study, Johnson et al. [56] also found that the children not exposed to a motivational climate showed minimum improvements in locomotor skills, but no changes in object control skills at the post-test. Although the previous studies only used autonomy support strategies to encourage children to engage in the FMS intervention program [27,32,55,56], the results of those studies produced the same outcomes in FMS competence as this study did. Nevertheless, from the SDT perspective, student learning is more successful when information is presented in a need-supportive way (basic psychological needs for autonomy, competence, and relatedness) [30,31,57]; thus, it is effective to apply need-supportive instruction to increase students’ motivation and achievement. Overall, this finding may strengthen the idea that promoting a motivational learning climate plays an essential role in helping children engage more in learning FMS.

Where our study differed from the previous studies [27,55] was that this study investigated physical activity behaviors during school and showed significant pre/post changes in their MVPA among the children in the need-supportive FMS group, but the children in the control group reported decreased physical activity behavior during school. This finding may indicate that a need-supportive, FMS-based afterschool program contributes to children’s physical activity behavior [7], while our participants already showed high physical activity engagement in school from the baseline (>2 h of MVPA during school time) compared to the general population of sedentary children (<1 h of MVPA daily [58]). As children are becoming more sedentary and engage in less physical activity during school [12], such findings may suggest that an emphasis on learning and practicing FMS with need-supportive instruction in the afterschool program should be established to improve children’s FMS competence and physical activity during school hours [59,60].

### 4.2. Gender Differences in FMS Competence and Physical Activity Behavior during School

The FMS intervention with need-supportive instruction enabled both boys and girls in the intervention group to gain substantial locomotor and object control skills (see Figure 2). Although the boys outperformed the girls in object control skills at the post-test, the girls significantly improved their object control skills from the baseline to after the intervention compared to the boys’ gain in object control skills. Compared to previous instructional motor skill intervention studies [22,61,62], this study demonstrated that boys’ object control skills were significantly higher than those of girls after the need-supportive, FMS-based afterschool program. However, both studies of Altunsöz and Goodway [61] and Goodway et al. [22] examined only the effect of an instructional motor skills intervention program among preschoolers (i.e., 3–5 years old). This study applied the FMS intervention for K–2nd grade students in elementary settings (6–8 years old); therefore, the finding that girls’ object control skills were lower than boys’ might be different due to the different age groups. One possible explanation for this finding is that girls tend to perform with lower competence in object control skills when they enter elementary school [5].

The effect size of the girls’ gain score in object control skills was comparable to that of the boys in this study. This outcome, which contradicted the findings of previous studies [22,62], might be due to the different instructional strategy in the motor skill program. This finding may be associated with the notion that need-supportive instruction could provide boys and girls with equally positive feedback, reinforcement, and encouragement of students’ motivation, as well as greater support to engage in the motor skill program [62]. Implementing need-supportive instruction in the motor skill program in this study could support children’s engagement in the motor skill intervention. As Barnett et al. [25] suggested, if girls can receive beneficial instruction and engage in learning experiences in an appropriate environment, gender differences in object control skills could be minimized. Thus, implementing need-supportive instruction in the FMS program may be the key to promoting both boys and girls to engage in FMS competence equally.

Although the statistical results indicated no statistically significant differences in MVPA across genders, it is interesting that girls in the intervention group were notably more physically active than boys from baseline after the intervention when compared to the previous study examining physical activity levels by gender, indicating that boys are more physically active than girls during school [63]. It may be difficult to interpret the information because of our unique participants, who had more than two hours’ MVPA during school, whereas the general population of sedentary children engage in less than one hour of MVPA daily [59]. Nevertheless, in our study, both boys and girls in the intervention group greatly gained MVPA during school from the baseline to post-test. Similarly, FMS intervention studies also demonstrated the improvement in daily MVPA after short- or long-term FMS intervention [16,64]. The findings suggest that FMS development is associated with greater participation in physical activity.

Several potential limitations existed in this study. The first limitation of this study was a validity issue about the FMS-based afterschool program to evaluate the effect of need-supportive strategies in this study. As this study did not include a comparison group, such as FMS programs both with need-supportive strategies and without need-supportive strategies, it would be premature to suggest future practice at this point. Secondly, as a large number of girls participated in the FMS-based afterschool program in this study (68% of the intervention group were girls), the girls might have felt more comfortable engaging in the FMS intervention and showing their motor skills in front of their peers, which might have affected the girls’ active engagement in the FMS intervention and the findings of this study. Thirdly, the unequal number of children between intervention and control groups was also one of our limitations; therefore, future intervention studies need to consider balanced participants between the groups. Fourthly, the different sample size of girls and boys in the study to measure gender differences may have influenced the findings and limited the inquiry. Thus, future studies need to consider a gender balance when recruiting participants, although this is part of the nature of data collection. Fifthly, in this study, our participants reported higher physical activity levels, even in school, than the general population [59]; thus, it may be difficult to generalize the findings. Finally, this study sought to objectively measure children’s MVPA using accerometers, but we only measured physical activity behavior in school (8:00 a.m. to 3:00 p.m.) and during the week (Monday–Friday) due to practical limitations. Thus, it is imperative for future research to examine physical activity levels, including in/out of school and during the week/on weekends, because children’s physical activity can occur more outside of the school environment (e.g., at home and during leisure times in the community) and on weekends (Saturday–Sunday) [65].

## 5. Conclusions

The current FMS-based afterschool program with need-supportive instructional strategies offers empirical evidence to promote children’s FMS competence and physical activity during school. Notable improvements in FMS competence and MVPA during school were observed among both boys and girls in the intervention group compared to those in the traditional afterschool program as a result of this study. Considering the fact that previous studies only focused on FMS-based instructions without any theoretically guided implementation, the findings of this study contribute to the pedagogical literature by proving that effective instruction leads to greater development of children’s FMS. Moreover, including need-supportive instruction in a motor skills/physical activity program may minimize gender discrepancies in motor skill development. Accordingly, we also suggest that afterschool program staff and physical activity practitioners learn and receive training on need-supportive instruction through a teacher/practitioner education program. Accordingly, they may develop and implement need-supportive instructional strategies to provide equal opportunities for both boys and girls to engage in motor skill programs.

## Figures and Tables

**Table 1:** Twenty-four intervention sessions under need-supportive instruction based on three basic psychological needs.

#	Focused FMS	Need-Supportive Instruction
1	Running	***Autonomy*** Offering rationale (e.g., emphasizing the importance of learning FMS if they want to grow stronger and play sports) [37], Using non-controlling voices or inviting languages (e.g., “Could you/we try…?”) [38], Minimizing pressure (e.g., “It’s okay to fail. How about we try it together again?”) [39]. Providing challenging tasks/goals that stimulate students’ enthusiasm (“Can you knock down all of the bowling pins by using your best underhand rolling?”) [39,40].
2	Underhand rolling	
3	Jumping	
4	Striking	
5	Galloping	
6	Catching	
7	Sliding	
8	Kicking	
9	Hopping	
10	Dribbling	
11	Leaping	***Competence*** Providing clear and understandable guidelines, rules, and feedback toward students’ behavior (e.g., providing specific instruction and encouragement to learn each FMS) [41,42]. Offering instrumental help and support (e.g., offering equipment based on the FMS-based lesson plans) [43]. Giving immediate positive and instructional feedback (e.g., “Wow, your swing is so great when you hit the ball. How about one step forward with your front foot when you hit the ball. Let’s try it again. Look! So much better”) [39,44,45].
12	Overhand throwing	
13	Galloping & Sliding	
14	Catching & Kicking	
15	Jumping & Hopping	
16	Striking & Dribbling	
17	Running & Leaping	
18	Underhand Rolling & Overhand throwing	
19	Galloping & Sliding	***Relatedness*** Creating a cooperative peer environment (e.g., passing the ball to the partner by kicking; throwing the ball to the partner by underhand throwing) [46]. Interpersonal involvement with emotional support (e.g., affection, warmth) to students (e.g., pep talk to the children who failed the tasks) [47]. Investing a substantial amount of time, energy, and resources to engage with students (e.g., friendly talk with the participants before/after the lessons) [48].
20	Catching & Kicking	
21	Jumping & Hopping	
22	Striking & Dribbling	
23	Running & Leaping	
24	Underhand rolling & Overhand throwing	

Note. # = Session; FMS = fundamental motor skills; all sessions were implemented by need-supportive instruction.

**Table 2:** Descriptive statistics of the groups by gender from the baseline.

Variable	Intervention (*n* = 25)		Group Difference	Control (*n* = 11)		Gender Difference
	Girls (*n* = 17)	Boys (*n* = 8)	*t(p*)	Girls (*n* = 6)	Boys (n = 5)	*t(p*)
Age, *M* (*SD*)	6.41 (0.79)	6.37 (0.91)	−1.19 (0.23)	6.66 (1.63)	7.00 (0.70)	0.40 (0.69)
Anthropometry						
Height (cm), *M* (*SD*)	119.83 (6.83)	124.03 (6.97)	−0.15 (0.87)	118.76 (8.16)	124.96 (6.98)	2.01 (0.06)
Weight (kg), *M* (*SD*)	23.34 (3.04)	26.47 (6.30)	−1.10 (0.27)	27.33 (5.07)	24.72 (3.81)	0.90 (0.37)
BMI (kg/m^2^), *M* (*SD*)	16.25 (1.70)	17.04 (2.52)	−1.52 (0.13)	19.22 (1.63)	15.73 (0.69)	−0.66 (0.51)
FMS competence						
Locomotor skills, *M* (*SD*)	23.88 (8.19)	25.31 (6.43)	−1.93 (0.06)	32.08 (6.70)	26.90 (9.15)	−0.03 (0.97)
Object control skills, *M* (*SD*)	18.97 (8.79)	29.06 (9.76)	−1.29 (0.20)	24.75 (12.19)	29.80 (9.37)	2.65 (0.01 **)
MVPA, *M* (*SD*)	148.09 (37.14)	138.29 (35.86)	−1.02 (0.32)	150.36 (62.46)	185.28 (69.01)	0.46 (0.64)

Note: *M* = mean; *SD* = standard deviation; ** *p* < 0.01.

**Table 3:** Descriptive analyses of FMS competence and moderate-to-vigorous physical activity (MVPA) at the pre- and post-test based on group and gender.

Variable	Intervention						Control					
	Girls (*n* = 17)			Boys (*n* = 8)			Girls (*n* = 6)			Boys (*n* = 5)		
	*M*	*SD*	*g*	*M*	*SD*	*g*	*M*	*SD*	*g*	*M*	*SD*	*g*
**Locomotor Score (Range 0–48)**												
Pre	23.88	8.19	1.55	25.31	6.43	2.34	32.08	6.70	0.17	26.90	9.15	0.25
Post	35.23	6.32		38.37	4.56		30.58	10.43		30.00	14.29	
Gain	11.35	6.54		13.06	5.49		−1.50	5.72		3.10	7.01	
**Object Control Score (Range 0–48)**												
Pre	18.97	8.79	1.92	29.06	9.76	2.02	24.75	12.19	0.10	29.80	9.37	0.03
Post	35.41	8.29		44.00	3.69		26.00	11.24		29.50	10.27	
Gain	16.44	5.19		14.94	6.32		1.25	9.22		−0.30	6.45	
**MVPA (mins)**												
Pre	148.09	37.14	0.49	138.29	35.86	0.81	150.36	62.46	0.13	185.28	69.01	0.26
Post	168.64	45.93		166.75	33.60		142.52	52.03		170.35	41.37	
Gain	20.55	27.77		27.46	24.71		−7.84	29.25		−14.93	30.58	

Note. *M* = mean; *SD* = standard deviation; *g* = Hedge’s *g*.
